# Case of Stricture of the Rectum—Operation and Cure

**Published:** 1849-09

**Authors:** W. C. Cole

**Affiliations:** Detroit, Michigan


ART, III.— *Case of Stricture of the Rectum — Operation and Cure.* By W. C. Cole, M. D., of Detroit, Michigan.

Mr. S——, aged 45, a jeweller by trade, of strong constitution, residing in Ohio, had been treated, while in Maumee City, for some disease, and among other things, stimulating injections were used, which denuded the rectum of, at least, its mucous membrane, and, probably, injured materially the muscular coat.* After the immediate imminent peril of his distressing situation was passed, a profuse suppuration was kept up for some six months, and when, ultimately, cicatrization was completed, it was only to leave him in a situation to loathe his very existence; for a permanent bridle had formed, five inches above the sphincter ani, around the entire parietes of the intestine, leaving only an aperture in the centre, large enough to admit the point of a goose-quill; and that so firm that it would not admit of the least dilatation.

In this condition he lived on, half starving himself, for two and a half years, never having a dejection, except by taking some hydragogue cathartic, or producing a perfectly fluid state of the feces, by injecting tepid water, &c.

Early in 1847, he consulted me. After hearing the history of the case, and examining the seat, and nature of the obstruction, I at once advised the division of the bridle, by the knife. His apprehensions, however, as to the consequences, led him to seek other counsel.

Accordingly he visited Columbus and Cleveland, when he was advised to try dilatation; but being convinced, from his own knowledge of the unyielding nature of the obstruction, that dilatation could afford him no permanent relief, on the 13th of May, 1847, he returned for the operation, which was practiced in the following manner:

The patient was placed on his back upon a sofa, with his legs drawn up. The index finger of the left hand was introduced into the rectum, the point of which was made to cover the aperture; then, gliding along, with its point on the finger, a common hernia bistory, which was readily passed

---

* The statement that these results were produced by the nitrate of silver seems gratuitous.—Editor.

through the small opening in the partition, and now, turning its edge against the bridle, it was freely divided both anteriorly and posteriorly.

The wound bled quite freely, but not dangerously, and the patient was left with no other directions than that he should remain quiet for a few days, and encourage, at least, one movement of the bowels every twenty-four hours. In two weeks he called to see me, and stated that he was perfectly well, that the soreness which followed the operation had entirely subsided, and that his bowels moved regularly, and without the least obstruction.

I have often seen this gentleman since the operation, and he has not even been troubled with constipation.

Detroit, August 15, 1849.

